# Proceedings of the American Dermatological Association at the Third Annual Meeting

**Published:** 1879-10

**Authors:** James Nevins Hyde


### Society Reports.

### ARTICLE IX.

### PROCEEDINGS OF THE AMERICAN DERMATOLOGICAL ASSOCIATION AT THE THIRD ANNUAL MEETING, HELD AT THE PARK AVENUE HOTEL, NEW YORK, AUGUST 26TH, 27TH, AND 28TH, 1879. (Reported by James Nevins Hyde.)

TUESDAY MORNING, AUG. 26TH. — At the conclusion of a business meeting, held with closed doors, the morning session was opened with the president, Dr. L. A. Duhring, of Philadelphia, in the chair. Present: Drs. J. C. White and E. Wigglesworth, of Boston; Drs. L. D. Bulkley, R. W. Taylor, H. G Piffard, C. Heitzmann, F. P. Foster, G. H. Fox and S. Sherwell, of New York; Drs. L. A. Duhring and A. Van Harlingen, of Philadelphia; Dr. I. E. Atkinson of Baltimore; Dr. W. A. Hardaway, of St. Louis; and Dr. J. N. Hyde, of Chicago.

THE PRESIDENT, Dr. Duhring, then read his address, entitled:

THE RISE OF AMERICAN DERMATOLOGY.

In tracing the history of the science, he gave an exhaustive résumé of all the earlier literature on the subject, and afterward a chronological account of the establishment of all chairs devoted to the subject in the principal colleges and special institutions for skin disease, as well as of the skin departments of the various large hospitals and dispensaries. Among the works referred to are the following: "A Brief Guide in the Small-pox and Measles," by Thomas Thatcher, which appeared in 1677, and is believed to have been the first medical book ever published in this country. The author of it was well known, not only as a learned physician, but also as a divine. A work on cancer by

Lieutenant-Governor Cadwalader Colden, a man of great learning and high attainments, and also author of a volume on the Climate of New York, which is better known than the other. "An Essay on the Causes of the Different Colors of People in Different Climates," is a long and elaborate treatise by John Mitchell, of Virginia, which is said to have been the first work ever published on that subject. Dr. Mitchell was also the author of a brochure on yellow fever as it prevailed in Virginia in 1741. "An Essay on the Causes of the Variety of Complexion and Figure in the Human Species," by Samuel Stanhope Smith, President of the College of New Jersey (1877). The book attracted a considerable amount of attention in Europe, and contained an account of the case of the negro, Henry Moss, of Maryland, who during a period of twenty years underwent a change of color from a deep black to a clear and healthy white. "An Experimental Dissertation on the Rhus Vernix, Rhus Radicns, and Rhus Glabrum," an able and exhaustive work by Thomas Hanfield. A "Dissertation on Perspiration," by James Agnew, of Princeton, New Jersey (Philadelphia, 1800), an essay of unusual excellence. "On the Warm Bath," by Dr. Lochette, of Virginia. "The Lues Veneria: the Modus Operandi of Mercury in curing Gonorrhœa," etc., by James Tongue, of Maryland, in which the author endeavored to prove that syphilis and gonorrhœa were two distinct forms of disease. "A Prospect of Exterminating the Small-pox," and a paper on the Cow-pox Virus, by Dr. Waterhouse, of Cambridge (1800). "The Jennerian Discovery," by C. R. Aiken. "The Structure of the Skin, with a View to the Diagnostics and Cure of Diseases usually denominated Cutaneous," a Boylston prize essay by George S. Shattuck, of Boston.

On the second of June, 1836, the "Broome Street Infirmary for Diseases of the Skin" was established in New York, with Drs. H. D. Bulkley and John Watson as physicians in charge. The following year a course of lectures on Dermatology was given at the Broome Street School of Medicine, by Dr. Bulkley, who had fitted himself for the work in Europe, and this was the first ever delivered in America on this subject. About the year 1830 the Medical Department of the University of Pennsylvania came into possession of a large and fine collection of models of skin disease, which had been purchased in Europe by Prof. George B. Wood, and this was the first treasure of the kind that was ever brought to this country. In 1861 Dr. J. C. White, the honored ex-president of the Association, gave the first course of lectures on Dermatology in Harvard University, and shortly after the close of the war chairs in this department were established in all the medical colleges of New York, Chicago, Philadelphia and other large cities. The New York Dermatological Society, an organization which has exerted so marked an influence on the study of skin disease, was founded in 1869, with Dr. H. D. Bulkley as its first president.

At the conclusion of the reading, a vote of thanks was unanimously adopted in view of the laborious research of the author and its valuable results.

DR. I. E. ATKINSON then read a paper entitled, A CASE OF INCOMPLETE VITILIGO. The reader, citing an earlier and similar case of semi-albinismus, reported by Beigel, proceeded to give the history of a mulatto woman, 25 years of age, a prostitute, measuring, in height, 171 centimeters; around the chest, 166 centimeters; about the umbilicus, 114 cm.; around the buttocks, 132 cm.; and around the thigh, 74 cm. At the age of 14, she had begun to menstruate, at 17 had ceased to be a virgin, had suffered from no other form of venereal disease than acute vaginitis, and was temperate in her habits. Five years previously, she began to exhibit the phenomena of the disorder on the back of the right hand, and subsequently was similarly affected upon the buttocks and the anterior surface of the trunk. Later, the surface of both hands and of the neck was irregularly involved.

The discoloration occurred in different shades, in discrete and confluent patches measuring one centimeter in diameter, each patch with a light center, enclosing irregular islets of integument. Usually the extensor surfaces were not involved. Over the breasts there were few patches, but occasional atrophic striæ. The surface of the abdomen and waist was exempt. There were atrophic striæ upon the anterior surfaces of the thighs, which posteriorly displayed multiple spots. About these there was a distinct increase in the pigment of the skin, but, as usual in these cases, it was difficult to note the difference. There was in no part desquamation, and sensibility was quite unimpaired. There were few hairs and these not altered in color. In December, it was clear that the patient was correct in stating that the patches appeared and disappeared. Then the hands were uniformly and symmetrically covered. There had been a restitution of normal coloring. The case was remarkable for: 1st. The complete absence of definite coloring matter in the affected patches. 2nd. For the complete recession of the disease. A similar case of reappearance of the normal tint had been reported by Dr. T. F. Wood, in 1877, in the case of a patient, nine years old, the folds of the skin being affected about the neck, though the disease commenced at the preputial fold.

DR. SHERWELL had seen somewhat similar appearances in a child of parents who were Carib Indians, the case being diagnosticated as one of macular leprosy. A photograph of the patient, taken by Dr. Fox, was exhibited.*

DR. WHITE referred to the fact that it was generally believed among the negroes that there was complete retrogression of the disease (vitiligo) and that this was remarkable in view of the absence hitherto of an exact record of such fact.

DR. TAYLOR stated that he had made such an observation in the person of a physician in Vermont, whose name he gave, who suffered from vitiligo of both hands only, during each summer, and who was quite free from the trouble in winter. Reference was made also to the series of cases collected by Dr. Yandell, of Louisville, all negroes, photographs of whom were in possession of several members.

DR. VAN HARLINGEN believed that in all these cases there was first an increase in the deposit of pigment. Hence the frequency with which the patches were noted among the races having dark skins.

* This little patient was subsequently exhibited to a number of members of the Association, who had met by agreement in the amphitheatre of the New York hospital for the purpose of examining patients affected with certain rare forms of skin disease. It seemed to be the opinion of the greater number of those present, that the case was one of leprosy, though not macular leprosy. The surface of the skin was irregularly dyschromic, and the clearly defined dark patches were distinctly elevated above the general surface of the integument.

DR. TAYLOR observed that he had seen leucoderma in an Indian.

DR. FOX said that cases of the character under discussion, were explainable by a reference to the incompleteness of the pigmentary change. In these incomplete forms of leucoderma, there was appearance and disappearance of the pigment. He pointed to the well-known difficulty of declaring that any patch upon the skin was absolutely pigmentless. In this disorder there was a coincident melanoderma and leucoderma, the former always preceding. To this succeeded the leucodermatous patches. With reference to the case of Wood, cited, he remarked that in the negro the inner surface of the prepuce was pigmentless.

DR. WHITE suggested that it would be interesting to know whether there was any change in the color of the parts in the instance of the circumcised negro.

DR. J. N. HYDE, then read a paper entitled, A CONTRIBUTION TO THE STUDY OF THE BULLOUS ERUPTION INDUCED BY THE INGESTION OF THE IODIDE OF POTASSIUM. He reported the case of an eight-months old infant, affected with an ordinary eczema capitis, which disease had been followed by an abundant crop of furuncles. For these the drug had been administered in 0.33 gr. doses for one month, with the result of producing a bullous rash, chiefly disposed about the ankles and wrists. The lesions were pin-head to pigeon's egg in size, and were vesicular, pustular and bullous in character. They contained an inspissated serum, for the most part, which did not freely exude when the mature lesions were ruptured. The latter were of a dark purple shade and did not fail to disappear a few days after the suspension of the remedy, passing through the phases of crusting and healing beneath a scab. The reader had annexed a table of all the recorded cases of this eruption following the administration of the iodide of potassium, including the cases reported by Bumstead, R. W. Taylor, Duhring, Tilbury Fox, Cazenave, Otis, Hutchinson and the reader. In concluding, he set forth the several reasons for dissenting from the opinion given by Dr. Tilbury Fox, that the eruption was essentially a sebaceous gland disorder, among them the fact that the bullæ had been seen by both Dr. Duhring and himself upon the palms of the hands, where Biesiadecki and others have shown that there are no sebaceous glands; the occurrence of blood in the blebs as reported by authors also pointed to another seat of the lesion.

Dr. White enquired whether the reader intended to exclude also the sudoriparous glands as the site of the disease, and

Dr. Hyde responded that he had no such intention, the clinical facts showing the improbability of a sebaceous origin having been suggested by the paper of the late lamented Dr. Fox, who had stated in so many words that the contents of the bullæ were altered sebaceous gland secretion.

Dr. Taylor remarked that he had seen a patient affected with tinea versicolor, to whom the iodide of potassium had been administered and whose shirt had been stained blue in consequence.

Dr. Wigglesworth had seen a patient in whom a similar eruption had been induced by the ingestion of the potassium bromide, taken under the orders of Dr. Brown-Séquard. When examined, there were traces of a pre-existing eczema of the legs. But bullæ were also to be seen, acuminate, of the size of a pea to that of the third joint of the middle finger, fringed about the edge, with deeply ulcerated base, a few containing blood. After rupture, there was a dark crust, indicating the presence of blood. The lesions were not accompanied with much itching, and occurred upon the leg and thigh and the regions below the knee and the ankle, being in places confluent. In other places the tissues were denuded to the extent of several square inches. When the drug was discontinued, the eruption disappeared rapidly, and had never recurred. In all, the eruption had lasted some three or four months. In response to a question, the speaker stated that no arsenic had been employed to moderate the severity of the symptoms.

Dr. White stated that Dr. James Putnam, of Boston, had secured excellent results by administering arsenic for the bromide of potassium eruption, the rash rapidly disappearing after the patient was under the effect of the arsenical preparation.

Dr. Taylor believed that arsenic controlled the rash produced by the iodide of potassium more readily than that induced by the bromide. In two cases treated by him where arsenic had been administered for relief of the bromide rash, it had seemed to have but little effect.

THE PRESIDENT stated that in his experience arsenic possessed very decidedly, a power of controlling the bromide rash. He recalled the case of a lady who came to him from Dr Seguin, of New York, with the eruption prominent over the face. She presented a note from Dr. S., in which it was stated that it was impossible for the time to discontinue the potassium bromide. Fowler's solution in doses of m.ij–iij., given with the bromide, procured almost immediate disappearance of the eruption. The improvement continued for two or three months, when the disorder recurred and was relieved a second time by the addition of the arsenical preparation. The treatment had been first suggested by Dr. Eccheverria of New York, some ten or fifteen years ago. Dr. Weir Mitchell of Philadelphia, had stated to the speaker that he had frequently employed arsenic for a similar purpose with the happiest effect.

Adjourned.

TUESDAY AFTERNOON SESSION.—DR. L. D. BULKLEY read a paper entitled, TWO CASES OF CHANCRE OF THE LIP, PROBABLY ACQUIRED THROUGH CIGARS. The reader began by referring to a history, given some years, ago of a young man communicating syphilis to a young woman who finished with her lips the end of the cigars which the former rolled together by the aid of his mouth. He was affected with buccal mucous patches. In both of the present cases the patients were physicians. The first, Dr. J., was thirty-three years old, married, the father of two children, and free from prior venereal accidents. Three weeks before the occurrence of any trouble, he had scratched his upper lip. When examined, he exhibited a clean-cut, indurated lesion at the site of the scratch, which had existed for six weeks. There were mucous patches in the vicinity, and a lesion upon the other side of the lip. A general macular syphilide followed, with rheumatoid pains. He had had no anti-syphilitic treatment. A diagnosis of chancre of the lip was made, and substantiated by Dr. F.

N. Otis, who saw the patient. He was a smoker of cigars and had never smoked a pipe.

The second case was that of Dr. F., who had suffered for from ten to fifteen years from a fissure of the lip. Six weeks before the consultation, the lesion gave origin to a serous discharge, and the glands of the submaxillary region became involved. The sore had been touched with a solution of chromic acid, 6.00 gr. to 30 gr., after this, nitric acid had been applied. In three weeks, the sore increased from the size of a finger-nail to that of a goose's egg, and an eruption ensued which first appeared over the forehead and which subsequently became general. When examined, the sore existed on the right of the median line and was 12 cm. in diameter. This patient was also a smoker of cigars; and remembered having, at about the time of the supposed infection, accepted and smoked a cigar, given him by a patient affected with syphilis of the mouth.

DR. HEITZMANN stated that this patient had consulted him also, and that at the time of his examination, he saw merely a swollen lip, covered with a dry crust. There was adenopathy of the right submaxillary region. He advised a poultice with the application of some cold cream to soften the part, and after the application, it was clear enough, the crust being removed, that there was a subjacent ulcer having all the appearance of a soft chancre. The patient had also a soft chancre upon the prepuce, which Dr. Bulkley had omitted to mention. Hence, the speaker had no thought of syphilis to follow, and had suggested that probably the submaxillary gland would suppurate. In fourteen days, the syphilitic eruption appeared. In view of all the facts, he thought that the doctrine of the so-called duality of the chancrous virus was no longer tenable. In one instance he had seen a penny-sized crateriform ulcer of the lip, which was supposed to have come from infection with a dentist's instrument.

DR. WHITE did not think that proof had been adduced sufficient to establish the fact of infection by the medium of cigars. Attention was then called to the fact that the reader had placed the word "probable" in the title of his paper.

DR. ATKINSON. briefly described some extra-genital chancres seen by him. In one case there was an abruptly elevated, red, glazed plateau of cartilaginous hardness on the right cheek. The hair there had fallen out, and the mouths of several follicles could be distingished as bloody points. There was co-existent submaxillary adenopathy and no history of venereal exposure. Secondary syphilis followed. In another case, a young man had a sore upon the finger as large as a ten-cent-piece, elevated, red, glazed, and of cartilaginous hardness, with epitrochlear adenopathy. A red streak was finally seen running up the arm, and a poultice applied. Quinine was administered internally. Phlegmonous erysipelas of the upper arm followed, and is now in process of healing. A sore of similar characteristics appeared on the dorsum of the thumb of an Irish woman, without destructive ulceration, red, glazed, and scantily discharging. No adenopathy.

DR. TAYLOR described two cases in which a cup and a towel had been the media of communicating syphilis. In the first case, a sailor, limited to the companionship of his vessel, had his lip split open with a pin and subsequently drank from the same cup with a mess-mate who had buccal mucous patches. The patient had been seen also by Dr. Otis. In the second case, a man had been confined to the same room with a syphilitic friend, and had used the same towels. In all cases of chancre of the lip, there was an ivory-like hardness of the pyramidal lesion, but he had observed the induration was much less marked in children, especially toward the vermilion border of the lip.

Responding to Dr. Heitzmann's remarks respecting the duality of the chancrous virus, he said that no one, today, attempted to diagnosticate a chancre by its external appearances alone, and that appearances were always fallacious. Soft chancres of the cephalic region had been observed by Morgan, Hutchinson, Bumstead, and others. In Jullien's new work, there was a table of the recorded cases.

DR. HYDE remarked that he had lately observed a series of cases of extra-genital sores of a syphilitic nature; one on the tongue of a railroad conductor, who smoked the pipe of his syphilitic brakeman; two, on the fingers of physicians, who had been probably inoculated through the medium of the contacts incidental to obstetric or gynecic manipulations; one, on the finger of a young woman who had cut her hand upon a piece of glass while attending an hereditarily syphilitic child. In all, there was marked induration, of the character described by the other speakers. But, with reference to the method by which these inoculations were effected, he had rarely found that the exact proof was at hand to establish the fact. In the vast majority of extra-genital chancres, it is simply impossible to secure proof as to the medium of infection. As to the existence of cephalic soft chancres, Dr. Taylor was probably aquainted with the interesting cases described some few years ago by Prof. Profeta, of Palermo, and published in the *Annales de Dermatologie et de Syphiligraphie*, the characteristics of the sores there described differing greatly from the appearances given by Dr. Heitzmann. In one instance, there had been a suppurating adenopathy of the submaxillary glands, the case of a butcher who had become infected in an attempt to minister to one of his customers similarly affected. The speaker did not think that Dr. Bulkley had established the fact of infection by cigars.

DR. VAN HARLINGEN described the case of a patient who had a sloughing ulcer follow excision of the tonsil, and subsequently developed papular syphilides which resembled flea-bites. Leeches had been applied to the throat, and the leech-bites took on the same appearance. Another instance occurred to him, where a physician had been infected in practicing insufflation of the lungs of a newly-born and asphyxiated infant. The child died in a few weeks; and it was found that its father and mother were syphilitic. Papular syphilides appeared upon the skin of the physician.

DR. FOX exhibited some photographs of patients with skin diseases; one designated as macular leprosy, of which mention has been made, the other a case of leucoderma, in which the disease disappeared in the winter, the white spots remaining.

He continued by reading a paper, entitled THE TREATMENT OF ECZEMA AND ULCERS OF THE LEG BY AN ELASTIC TUBULAR BANDAGE.

The reader described the use made by him of a straight rubber tube, measured when lying flat, slipped over the limb so as to embrace it. It exerted pressure chiefly over the calf, but compressed also other parts, so that, for example, in eczema madidans and ichorosum, the serum was pressed from out the tissue. This serum macerated the parts, and, if the bandage was worn for too great a time, it removed the epidermis. The rubber naturally also afforded support to the limb. Being light, it possessed advantages over the other bandages in the market, and was also cheaper. The limb was first oiled, and the bandage then applied directly over the inflamed surface. In three days, ordinarily, an effect could be produced equal to that obtainable by other impermeable dressings in three weeks. Then should follow one of the usual topical medicaments, such as the ointment of the zinc oxide. The reader showed several bandages, of various thickness, one upon his own leg, which he had worn for a day, and that, during a walk four miles in length. When the bandage was removed, he showed a slight moistening of the underlying integument. He also exhibited photographs of patients affected with eczema of the legs, before and after the wearing of the tubular band. He believed that it would prove of value also, in the treatment of varicose veins of the lower extremities, acting upon the principle first suggested by Mr. Erichsen, a simulation of the action of the valves of the veins. In ulcers with callous edges, it would answer the indications for treatment in a remarkable manner. The advantages claimed were enumerated as follows: 1. Lightness, producing gentle pressure and a trifling degree of fluid transpiration. 2. Equability of pressure, as the tubular band fitted like an extra epidermis. 3. The saving of time and trouble in its application; it scarcely required removal until its effects were produced. 4. Cheapness.

DR. BULKLEY suggested that it might be applied also to the hand.

DR. SHERWELL suggested that it might also be manufactured with perforations, and a trap for the heel.

DR. FOX responded that he had tried several of these suggestions and had not found the results satisfactory. The use of the Martin bandage in eczema, required that it be applied above the knee, in order to effectually retain its position, while the tubular bandage suggested by him, could be made to cover any portion of the limb. It also produced much less artificial eruption. In cases, a starch powder was needful as a preliminary dressing. DR. HARDAWAY stated that he had seen the induced eczema, following the use of the Martin bandage, extend in a disagreeable manner.

DR. BULKLEY called attention to the fact that Dr. Martin employed a bandage of a particular manufacture, and not indiscriminately those made of rubber.

DR. C. HEITZMANN then read a paper entitled MICROSCOPICAL STUDIES ON INFLAMMATION OF THE SKIN, of which the following is a full abstract:

On looking over the history of the doctrine of inflammation in general, we are struck by the fact that since the date of the first use of the microscope, repeated revolutions have occurred. In the fifth decade of our century the so-called "humoral pathology" was the leading doctrine—its founder, the late C. Rokitansky, of Vienna, by whom the whole process of inflammation was thought to be caused by an anomalous mixture of the blood, and to run almost exclusively in the vascular system. Even the newly formed elements, the so-called "exudation and pus-cells" were considered as products of the fluid exudation, almost nothing being known about the changes in the inflamed tissues themselves. At that time the process of inflammation was demonstrated with the low powers of the microscope on the web-membrane of the frog. In the sixth decade the "cellular pathology" became the leading doctrine, and its chief representative, R. Virchow, of Berlin. This doctrine neglected the blood vessels, and their products, viz., exudation, to such an extent, that, based upon observations on cornea and cartilage, in which avascular tissues inflammation could be produced, the blood vessels were thought to be irrelevant factors of the process of inflammation. In the seventh decade, I. Cohnheim, of Leipzig, made the assertion, based upon observations upon the mesentery and the tongue of the frog, that in the course of inflammation the colorless blood-corpuscles almost exclusively participate, through emigration from the blood-vessels (capillaries and small veins), while the so-called cells of the tissues simply perish. In this view, all newly appearing elements, the inflammatory new formation and the pus-corpuscles, were emigrated blood-corpuscles.

Each of the leading doctrines had in its time a crowd of followers and believers. Rokitansky himself adopted in turn, in its main features, the cellular pathology, which holds good even in our day, for the majority of pathologists, so much so, that the emigration theory in its full perfection was taken up but by a few observers, some of whom, however, admit that, beside the colorless blood-corpuscules, the "cells" also of the tissues share in the inflammatory process.

Toward the end of the last and the beginning of the present decade of our century, S. Stricker, of Vienna, was the strongest opponent of the emigration theory, asserting that in each of the former doctrines there was a partial truth. Stricker studied inflammation mainly in the cornea, and the conclusions he reached were that blood-vessels and nerves are necessary participants in the inflammatory process, that the inflammatory new formation is almost completely a product of the "cells" of the tissues and their off-shoots, and that the emigration of the colorless blood-vessels is beyond the proof of our present methods of observation, so far as its participation in the inflammatory new-formation is concerned. Stricker was the first to prove the correctness of the hypothesis of John Hunter, that the essential changes in inflamed tissue consists in its reduction to a juvenile, embryonal condition.

Such was the standing of the doctrine of inflammation when I took up its study in 1872, in Vienna. I chose, first, cartilage, which is by no means an avascular tissue, as was thought in former times, but is provided with medullary spaces, which contain a complete vascular system, though in relatively great distances from each other, to such an extent that large territories of cartilaginous tissue are devoid of blood-vessels. Next I studied bone, which is far better fit for this purpose than any other tissue, and by degrees I extended my observations over pretty nearly all the tissues and organs of the animal body, including also the complicated organ termed skin. The results of these observations are laid down in a number of publications, both in German and English, partly from the pen of gentlemen of the medical profession, who have worked in my laboratory under my surveillance. I can fully corroborate the assertions of S. Stricker, above quoted. At the same time I take a step forward, and farther than any previous investigator; so much so that I venture to say, the inflammatory process is perfectly plain to-day in its minutest features, so far as our best modern microscopes allow of a definite conclusion. More than that, the present results of research are in full accordance with clinical observation; an accordance which was impossible when all former doctrines were accepted. My researches also explain the constitutional influence upon the inflammatory process in quite a satisfactory manner.

Let us briefly recapitulate the minute anatomy of the two main tissues, entering the structure of the skin, viz., epithelium and connective tissue. Each fact has been demonstrated for nearly five years to a large number of attendants in my laboratory, and proved by numerous observations on the tissues named, both in their normal and morbid conditions. The epithelium represents a continuous layer of living matter on the surface of the body, and on all cavities and elongations which are in direct or indirect communication with the outer surface. The elements of this layer are protoplasmic bodies, flattening each other, separated from each other by a cloak of horny cement-substance, and uninterruptedly united with each other by means of delicate spokes, the formerly so-called "thorns," traversing the cement-substance. The living matter, which produces a delicate reticulum in each protoplasmic body, its points of intersection being termed nucleoli, nuclei, and granules, traverses the cement-substance in the shape of "thorns," and thus produces continuity through all the living layers of the epithelial elements, as well as with the underlying layers of the connective tissue. Epithelium is devoid of blood and lymph vessels; but when living, is supplied with a large amount of nerves, which, in the shape of very minute, beaded fibers run through the cement-substance, and are here in direct connection with the fibrillæ of living matter, indirectly, therefore, with the reticulum of the living matter within the protoplasmic bodies themselves. Delicate excavations in the cement-substance, analogous to the well-known bile capillaries of the liver, and evidently destined to carry the nourishing material to the epithelia, have been recently discovered by Arnold of Heidelberg.

The connective tissue in that variety entering the structure of the derma, is built up by bundles of fibers, which, through manifold decussations produce a very dense feltwork, coarsest toward the subcutaneous fat tissue and finest in the outermost, so called, papillary layer of the derma. The main directions, in which the bundles of the fibrous connective tissue cross each other on different portions of the skin, have been accurately studied by C. Langer, of Vienna, and Tomsa, of Kiew. The bundles are bounded in many instances by a very dense basis substance, representing the elastic fibers, and separated from each other by narrow layers of a cement-substance (Tomsa), which in its chemical features is kindred to the glue-giving basis substance of the fibrous connective tissue in general. In this cement-substance there are imbedded delicate formations of protoplasm, greatly varying in amount in the derma of persons of different age. They represent formations analogous to nuclei, formerly so called "connective tissue cells," at present considered as compact masses of living matter, or delicate reticular layers of living matter, which with a power of 500 diameters of the microscope, look finely granular. The whole glue-giving basis substance of the bundles is traversed by a delicate reticulum of living matter, in direct union with all protoplasmic formations between the bundles, with all blood and lymph vessels, with all nerves, and with the columnar epithelia, nearest to the papillary layer. This reticulum of living matter, which I first discovered in 1873, is invisible in the fresh condition of the connective tissue, or in specimens obtained after hardening in alcohol or chromic acid solution, owing evidently to the refracting power of the basis substance; but can be brought distinctly to view through staining methods, such as nitrate of silver stain in a negative, and chloride of gold stain in a positive way, and through observation in all instances, when the refracting power of the basis substance is increased (deposition of lime salts) or decreased (liquefaction, dissolution) in different normal and morbid conditions of the connective tissue. Only the meshes of the network of the living matter contain the glue-giving basis substance, which, as the history of development of the connective tissue demonstrates, is produced by a chemical alteration of the lifeless protoplasmic fluid itself. The bundles of the connective tissue of the derma accompany all elongations of epithelial nature. The bundles produce the follicle around the root sheaths of the hair, the capsule around the sudoriparous glands, the accompanying layers around their ducts. The bundles of connective tissue are traversed in oblique direction by bundles of smooth muscle fibers, viz., the arrector pili, the numerous muscle bundles in the derma in and around the nipples, the scrotum and the labia majora. The connective tissue, furthermore, is traversed by relatively scanty blood vessels in the derma, by numerous capillaries in the papillary layer, and by lymph vessels, which produce a perfectly closed system, as demonstrated by Teischman, of Krakau, and Sappey, of Paris. Lastly, numerous nerves run through the connective tissue, both of the medullated and non-medullated variety. The former mainly produce the tactile corpuscles within the papillæ, the latter being partly sensitive, terminate in the epithelial layer ; partly motor, terminating in the bundles of smooth muscle fibers; partly vaso-motor, spun around the blood vessels (Tomsa); or secretory and trophic, supplying the sebaceous and sudoriparous glands, and all protoplasmic formations of the skin. About the termination of the latter varieties of the nerves, almost nothing is known.

The process of inflammation of the skin I have studied on specimens from a syphilitic papule; from small-pox; from an ulcerating sac of umbilical rupture in a cat; in its terminations on specimens of elephantiasis of the scrotum and the labia majora; as an accompanying process on the skin of the female breast in mastitis and cancer; on the skin covering different benign and malignant tumors, or directly engaged in the formation of such tumors; all types and varieties of which are represented in my collection. The results being almost identical in regard to the essential changes in the tissues of the skin, I can confine myself to the description of the inflammatory process in small-pox, of which I obtained six different specimens from the Blackwell's Island Hospital, amongst these, two of hæmorrhagic small-pox. In these specimens there are represented all stages of the disease in the most satisfactory manner.

The coarser microscopical relations in the formation of small-pox, have been accurately studied by Auspitz and Basch, of Vienna; the minute features, observable with high magnifying powers of the microscope—800-1200 diam.—only, and based upon the knowledge of the normal anatomy of the engaged tissues, are as follows :

First, the epithelial layer, termed rete mucosum, appears slightly thickened in circumscribed spots ; the swelling is due to a coarse granulation of the epithelia themselves. The coarse granulation is produced by an increase of living matter within the protoplasmic bodies, evidently through an augmented afflux of nourishing material in the stage of hyperæmia. The points of intersection of the network of living matter, formerly so called granules, become enlarged, many of the nuclei shining and solid, and at the same time the threads traversing the cement substance, the formerly so called "thorns," become thickened. The underlying papillæ are slightly enlarged in all diameters, partly owing to a dilatation and engorgement of their capillary blood vessels, partly through a peculiar change of the bundles of the connective tissue and the protoplasmic bodies between them. The latter look slightly enlarged, and in many instances coarsely granular, the former are partly transformed into protoplasm. In other words, where before there were present bundles built up by a glue-giving basis substance, at present the reticulum of the living matter, formerly hidden in the relatively solid basis substance, through a liquefaction or dissolution of this substance, became visible again. No other proof of the presence of an exudation in this stage is obtainable except the liquefaction of the gluey basis substance. This stage of inflammation is termed "papular." Next, in the midst of the papule, on one or on several spots, the exudation makes its appearance—the outer or epidermal layer at no time participates in the morbid process. In some epithelia we notice an enlargement of the meshes of the living reticulum, the latter is first stretched, afterward torn apart, the granules being now suspended in the liquid exudation. Where there were present epithelia before, a small, irregular cavity is visible. If several such cavities had formed in a papule through a continuously increased accumulation of the exudation and destruction of the epithelia, the separating layers of the epithelia become compressed and produce septa, traversing the cavities. Such septa greatly vary in number and width. The neighboring epithelia look very coarsely granular. Many of them have lost the inclosing cement substance, and are thus transformed into protoplasmic clusters, in which, through a considerable increase of the living matter, new shining lumps of different size have appeared still in continuity with the neighboring reticulum by means of delicate threads, the so called endogenous formation of new elements. The result of this process is the formation of an irregular cavity in the midst of the greatly widened rete mucosum, traversed by septa of compressed epithelia; filled with an exudation in which there are suspended numerous delicate granules, generally termed coagulated albumen, and a varying amount of irregular threads in the shape of a feltwork, the coagulated fibrin. Scanty protoplasmic bodies are suspended in the exudation also, perhaps remnants of the destroyed epithelia, perhaps immigrated inflammatory or colorless blood corpuscles.

In this condition of the rete mucosum also, the underlying connective tissue exhibits considerable changes. The papillæ have disappeared, evidently through the pressure from above. The transformation of the connective tissue into protoplasm has advanced, in some instances to such a degree, that the uppermost layers of the derma are replaced by numerous indifferent, or medullary or inflammatory elements, as a rule, clustered together. All these elements, however, are in interrupted connection with each other through delicate threads of living matter, fully analogous to those of the epithelia, and thus the inflamed tissue, though reduced into its medullary condition, still represents a tissue. The stage of the disease in which the changes just described have taken place, is known as the vesicular stage of small-pox.

Lastly, pus corpuscles appear in the cavity within the rete mucosum; they doubtless arise in their main mass from the epithelia, traversing and bounding the cavity. Through the increase of living matter in a large number of epithelia, shining lumps appear, first homogeneous looking, afterward through the intermediate stage of vacuolation transformed into nucleated protoplasmic bodies, with a fully developed reticulum of living matter—the pus corpuscles. The main source of pus corpuscles, therefore, are the epithelia themselves, the endogenous formation. How many of the pus corpuscles have appeared through an immigration from below, from the inflamed connective tissue or from the blood vessels, nobody can tell. The immigration is a sensible hypothesis only, without direct proof or foundation, while the endogenous formation can be directly traced in all its stages. The pus corpuscles look coarsely granular, viz., are supplied with a large amount of living matter on the points of intersection of the living reticulum in persons of a good strong constitution ; on the contrary, they are finely granular, that is, scantily provided with living matter in persons of a weak, so-called scrofulous or tuberculous constitution, or in persons debilitated by different acute or chronic diseases. In the former instance the pus is thick and yellow, in the latter instance watery, serous and pale. The subjacent connective tissue in many instances does not advance beyond its reduction into a medullary tissue. In some cases, however, the newly appeared and newly formed medullary elements, which produce the infiltration of the derma in a varying depth, are also torn asunder, and thus represent pus corpuscles, which commingle with the pus sprung from the epithelia, and share in the formation of the abscess.

This stage of inflammation is known by the term pustular stage of small-pox, and represents the typical termination of the whole process. The pustule either bursts or its contents dry and produce the crust. So long as the inflamed derma remains in the condition of medullary tissue, so long, therefore, as the medullary or inflammatory elements remain connected with each other, the reformation of a glue-giving basis substance in the shape of bundles of fibrous connective tissue will be accomplished, without the formation of a scar. If, on the contrary, a part of the connective tissue has been transformed into pus, and thus completely destroyed, the result will be a cicatrix. Mere epithelial suppuration heals without, suppuration of the connective tissue always with the formation of a scar. The pigmentation of the skin, so common after small-pox, is due to the imbibition by the reticulum of living matter of the epithelia, of the coloring matter of the red blood-corpuscles ; or by changes of directly extravasated red blood-corpuscles, both in the rete mucosum and the derma. Such extravasations occur in all severe cases of small-pox, in the highest degree, of course, in hæmorrhagic small-pox.

My observations on inflamed portions of skin have led me to the following conclusions:

1. In epithelium, the first step of the inflammatory process consists in an increase of the living matter, both in the protoplasmic bodies and between them; the former produces the coarse granulation of the epithelia, the latter the thickening of the so-called "thorns" in the cement-substance. Any particle of living matter, both in the epithelia and between them, through continuous growth, may lead to a new formation of the epithelial elements, with termination in hyperplasia of epithelium (psoriasis, squamous eczema, horny formations, etc.).

2. In connective tissue, the first manifestation of the inflammatory process is the dissolution of the basis-substance and reappearance of the protoplasmic condition; by this process and the new formation of medullary elements, which may start from any particle of living matter, the inflammatory infiltration is established. The sum total of the inflammatory elements, which remain united with each other by means of delicate off-shoots, represents an embryonal or medullary tissue. If the new formation of medullary elements be scanty, resolution is accomplished by reformation of the basis-substance (erythema, erysipelas, etc.). If, on the contrary, the new-formation of medullary elements be profuse, a new-formation of connective tissue will result (hyperplasia, scleroderma, elephantiasis, etc.).

3. Plastic (formative) inflammation may be accompanied by the accumulation of a larger amount of a serous or albuminous exudation in the epithelial layer (miliara, sudamina, herpes), or in the connective tissue of the derma (urticaria). In both instances complete resolution will ensue.

4. Suppuration in the epithelial layer of the rete mucosum is produced by an accumulation of an albuminous or fibrinous exudation, by which a number of epithelia are destroyed, and by new-formation of pus-corpuscles from the living matter of the epithelial elements themselves. Epithelial suppuration heals without the formation of a cicatrix (eczema madidans et putulosum, impetigo, pemphigus, variola).

5. Suppuration in the connective tissue of the derma results from the breaking apart of the newly formed medullary elements, which being suspended in an albuminous or fibrinous exudation, now represent pus-corpuscles. Pus is a product of the inflamed connective tissue itself, and always a result of destruction of this tissue. Suppuration of the derma invariably heals through cicatrization (abcess, furuncle, acne, ecthyma, variola).

The reader illustrated his paper by extempore sketches upon cards, which were handed to members of the Association, during the reading, representing the normal anatomy of the skin and each stage of the process described.

In response to questions by Drs. ATKINSON and SHERWELL, he stated his belief that the ultimate changes in the variolous pustule were the result of fatty degeneration of pus cells, resulting in a cheesy product. The cells, after absorption of serum, shrivelled from lack of nourishment. When hæmorrhage occurred, crystals of hæmatoidine were visible.

DR. HYDE inquired whether the reader accepted the usual explanation of the phenomenon of umbilication viz.: the tying down of the roof-wall by stretched rete bodies, and was answered in the affirmative.

Adjourned.

WEDNESDAY MORNING SESSION. — The following officers were elected for the ensuing year: President, Dr. L. A. Duhring; Vice-Presidents, Drs. E. Wigglesworth and W. A. Hardaway; Secretary, Dr. A. Van Harlingen; Treasurer, Dr. I. E. Atkinson. The time and place of the next meeting were decided to be the last Tuesday, Wednesday and Thursday of August, 1880, at Newport, R. I.

The first paper of the morning was read by DR. H. G. PIFFARD, and entitled VIOLA TRICOLOR. The reader cited several writers of the years 1772, 1779, 1785 and 1843, who described the wild pansy (*viola tricolor, pensée sauvage*), as incisive, vulnerary, valuable in certain diseases of the skin, and especially in in *crusta lactea.* He had employed specimens of the plant imported by himself, and exhibited these, together with several other specimens obtained at various drug establishments in the United States, the latter differing greatly in external characteristics, both from the imported specimens and from each other. The reader explained his use of the wild pansy, both in a fluid extract, prepared from his imported specimens, in infusion, and in combination with purgative doses of senna. His experience with it, led him to believe that it was chiefly valuable in eczematous disorders of the upper part of the body, and that it decidedly aggravated those occurring in the lower portions. For a time, even in cases of eczema capitis, there was an aggravation of symptoms under its use. When administered, the urine of the patient had the odor of the urine of the cat.

DR. WHITE inquired whether the article had been administered to individuals in perfect health, and whether the effects of the senna, administered in combination with it, had been duly separated.

DR. HEITZMANN proceeded to refer to the well known experiments of Hebra in testing the value of internal medication in skin diseases, by the administration of an infusion of hay, internally, which, in combination with local treatment, produced such wonderful effects as to give rise to the couplet:

"O! Gramen, Gramen!
What a powerful medicamen!" [Laughter.]

DR. FOX expressed his belief in the value of both internal and external medication in skin diseases, and thought that he had obtained results by that method, which could not have been otherwise produced. Purgation was certainly of value in diseases of the head. Hebra had, it was true, employed the viola tricolor as a placebo, but he himself had employed it with good results. As to the value of catharsis, he referred to a case of erythematous eczema of the face, where he had employed a lotion of carbolic acid to relieve the itching, which could not be made to improve until he had administered a mixture of sulphur and senna, when improvement rapidly followed. Hebra was certainly in error in condemning purgation.

DR. WIGGLESWORTH responded that Hebra recognized the value of purgation in diseases which were slowly progressing, but had laid stress upon the merely temporary value of such procedures. He cautioned against reliance upon internal treatment of the character proposed.

DR. HARDAWAY stated that he had been disappointed in departing from treatment which was not strictly local, but in certain cases of eczema had obtained benefit from the internal administration of ergot.

DR. SHERWELL thought a mild mercurial, in such cases, often proved of the greatest value.

DR. PIFFARD concluded the discussion by referring to the views of the French school; the eminent Professors Hardy, Bazin, Guibout, Devergie, and others, all employing and laying great stress upon internal medication. The best results were unquestionably obtained by a mixed treatment.

DR. VAN HARLINGEN then read a paper entitled, A CASE OF HITHERTO UNDESCRIBED TUBERCULO-PUSTULAR DISEASE OF THE SKIN. The patient, aged 39, had spinal curvature, but the general health was fair. There was no specific family history. The present disease was supposed to have appeared early in life, since which time there had been no relief. It was worse in winter, and had been at times accompanied by adenopathy, chills and diarrhœa. When examined, two skin disorders were recognized. 1st. A papular eczema of violaceous tint, affecting the arms, forearms, neck, cheeks, nose, brow and back, the scalp being free. It was a smooth eruption, in no places scaly. 2nd. Over the lower third of the thighs, the legs and the ankles, were solitary, large, finger-nail sized, roundish, firm, nodules or tubercules. They were semi-solid, firm, hard, elevated to the extent of 2 to 3 mm. and not acuminate. They were seated on a harsh, dry, and slightly infiltrated integument. Fifty were counted upon the left leg; 30 upon the right; 8-10 over the thighs. Their course was; First, itching, with an elevation resembling urticaria. In 12 hours, there was a fully-developed firm nodule, flattened like a mint-drop, of a dusky pink color. In one to two days, there was central exudation, with production of sero-pus, which finally became completely purulent. Absorption occurred last. If emptied, the evolution was more rapid. Then they assumed an ash color, and the lesions were smaller. Before complete disappearance occurred, weeks and even months elapsed. No cicatrix was left. Then a curious fact became apparent; in precisely the same localities, the same phenomena recurred, and in similar stages. There were no subjective sensations. Under the influence of the potassium iodide, a papulo-pustular eruption had at one time developed, for which the liq. picis alkalin. had been employed. Once, a small, tender, sausage-shaped tumor appeared on the inner face of the thigh, which was thought to be a scrofulous lymphatic development.

Microscopical examination of a section of a urticarial tumor, 24 hours old, showed the disease to be purely inflammatory. Under a low power, the stratum corneum was seen to be raised as in vesiculation, the epidermis being split, the cells of this layer not staining. Below, there was a normal rete with exudation granules, considerable pigmentation, and no small celled infiltration.

DR. HEITZMANN, to whom the sections had been submitted, remarked that the second specimen exhibited the ordinary signs of inflammatory infiltration in flat vesicles. The third specimen was especially excellent and interesting, exhibiting the large papillæ crowded with inflammatory corpuscles, the main changes occurring in the capillaries of the papillary layer. A number of these capillaries were transformed into solid strings, with a still-to-be-recognized endothelial wall. Pigment granules were abundant, as is often the case in organic disease. Atrophy had resulted from the pressure of the newly-formed connective tissue, so that the capillaries were changed into mere bundles of connective tissue. The pigmentation in these cases resulted from the narrowing of the vessels, the imprisonment of the blood corpuscles, and the subsequent changes by which the latter became broken up, the pigment coloring staining all the neighboring tissue. The case did not seem to him to have been an especial one. It was an eczema of long continuance, probably aggravated by scratching.

DR. VAN HARLINGEN called the attention of the last speaker to the rapid changes occurring in the part.

DR. DUHRING, who had seen the patient, did not think that the case was an eczema, and did not agree with the author of the paper in diagnosticating the eruption over the upper portion of the body as a papular eczema. The ring of pigment was distinct and peculiarly characteristic in all the lesions, and the recurrence of the papules in the exact site of their predecessors was a striking feature. He believed the disease to be one *sui generis*.

DR. ATKINSON believed that he had seen cases which presented features intermediate between eczema and those observed by the reader. They were of the character of erythema nodosum.

THE CHAIR (Dr. Hyde) inquired whether in those cases there had been recurrence at the exact site of former lesion, as in Dr. Van Harlingen's case.

DR. ATKINSON responded that he had not observed such recurrence.

DR. S. SHERWELL then read a paper entitled, TATTOOING OF NÆVI. In it he described the results of his method, as previously published, in the case of a lady who had a port-wine stain of the face, involving the vermilion border of the lower lip, extending to the facial artery, with ill-defined peninsular-like prolongations at several points. During a period of 18 months, he had twice tattooed over the whole with a mixed solution of chromic and carbolic acids, diluted; there had been a slightly flattened scar as the result. Seven months had elapsed since the last operation. He had been unsuccessful with the method of Balmanno Squire, of London. The instrument employed by him was shown, and he stated that he laid great stress upon the after treatment of the cases by the employment of pressure with a coat of collodion. As there was a great difference between the sensitiveness of different patients, he usually first performed a tentative operation. Dr. Sherwell then exhibited his patient to the Association.

DR. HEITZMANN remarked that the title of the paper contained a misnomer. "Tattooing" was the introduction of pigments beneath the skin, such as India ink, as used by oculists in the cornea, or cinnabar, to produce the vermilion border of the lip in cheilo-plastic surgery, the introduction of indigo, etc. The pricking or puncturing operation, proposed by the reader, had long been known. The end to be attained was simply the excitement of sufficient artificial inflammation to occlude the vessels. Pricking with the introduction of a foreign substance, electro-puncture, etc., had all been used. But the introduction of pins alone was sufficient to produce the result.

DR. SHERWELL responded that his claim was not as to the originality of the operation, but to the introduction of a foreign substance and to the subsequent collodion compression.

DR. HARDAWAY said that he had employed electrolysis in such cases with better results than by any other method. The destruction was immediate, the hæmorrhage nil, the scar slight. He employed a bundle of No. 13 cambric needles in a holder.

DR. FOX had employed electrolysis in one case with fair results. He was of the opinion that multiple punctures were better. He considered the results obtained by Dr. Sherwell remarkably good. The needles employed by himself were closely set and in a leaden handle, for convenience of use. In 1874, he had recommended a smaller instrument. The results obtained by him, after the method of Squire, had not been satisfactory. The production of complete freezing was very difficult, yet he had taken great pains in the three cases where he had tried it, and in all been disappointed.

DR. SHERWELL responded to a question, that he expected to complete the cure of his case after one further operation. He preferred few needles, as the area of impact was thus more evenly attacked.

DR. WHITE said that much was to be learned in the treatment of these deformities. He had tried Squire's method on two occasions and, failing completely, would never try it again. As to repeated acupuncture, he had never obtained the results of Dr. Sherwell, even in the superficial varieties known as "spider-cancer." We cannot obliterate all the telangiectasic spots. In none of the deeper-seated forms, would he expect to accomplish success. He had employed electrolysis upon one occasion with a single needle.

DR. HYDE had employed electrolysis with a single irido-platinum needle upon two occasions, and the results had been quite satisfactory, although in one case the nævus was small. As to recurrence, there had been a small pin-point sized vascular spot return in the centre of the seat of one operation, which he had watched for six months, and which in that time had not increased in size.

DR. WHITE then presented the ANNUAL REPORT OF THE COMMITTEE ON STATISTICS OF SKIN DISEASES IN THE UNITED STATES. From the seven districts assigned to members, five reports had been made, these including nearly 6,000 cases of skin disease, occurring in both dispensary, hospital and private practice. The Boston, New York, Philadelphia, St. Louis and Chicago districts were included in the report. Tables were presented in which the number of cases of each disease was set opposite the name of each, in the order of the classification adopted by the Association. A special report on the subject of leprosy was also made, in which were included the cases gathered by Dr. Saloman, of New Orleans, 14 in all; five cases reported by Dr. Hyde, one made the subject of a clinical lecture and observed by himself, four occurring in his district in the States of Wisconsin and Minnesota. Notes of these cases were appended. Also one under the care of the Chairman of the Committee, Dr. White, in Boston, the patient being a native of Louisiana.

DR. HEITZMANN, referring to the difficulty of diagnosticating leprosy, remarked that he had been summoned, with a physician, to see a case of "leprosy," which proved to be melanotic sarcoma.

DR. HYDE responded that the diagnosis in the Minnesota and Wisconsin cases had been made by Scandinavian physicians, in every case; one of them for years the pupil and hospital assistant of the late Professor Boeck, of Christiana, and all of them familiar with the disease as it occurs in their native land.

DR. DUHRING remarked that he had lately seen a well-marked case of the disease in a native of Cuba.

THE COMMITTEE ON NOMENCLATURE reported merely in favor of correcting a typographical error which appeared in the first copy of the classified list of diseases; and also in favor of changing the relative positions of carcinoma and sarcoma in class 6, sec. 3, leaving thus the word sarcoma in that class, and making a new sub-section, 4, in which the word carcinoma should appear. The changes were agreed to. On motion of

DR. HYDE, it was agreed to defer consideration of further changes in the classification and nomenclature until the next meeting, members to propose changes in writing to the committee before the date of the meeting; and the committee to be required to present such proposed changes to the association.

Adjourned.

WEDNESDAY AFTERNOON SESSION.—DR. HARDAWAY read a paper entitled A CASE OF MULTIPLE TUMORS OF THE SKIN ACCOMPANIED BY INTENSE PRURITUS. An unmarried woman, 51 years of age, complained that, 20 years ago, she first suffered from "blisters" upon the hands, which were accompanied by itching. The bullæ at first contained fluid, afterward, in the seat of each, a tumor appeared, which has since persisted. There was no cachexia, and her physical appearance was fair. The parts invaded were, the hands, arms, shoulders, feet and legs as far as the knees. The rest of the body was not implicated. The lesions were darkly pigmented, and varied in size from that of a large pea to a hickory nut; the largest, elevated five and more millimetres. The epidermis was thickened and roughened. Among the tumors were nodulated patches or plates, as large as a silver dollar, some of them long and narrow. The tumors preponderated upon the outer aspect of the arms and legs, the palms and soles not being spared. In parts, the eruption was quite painful, in consequence of the cracking of the integument. The pruritus was excessive, both in the tumors and plates, which moreover presented a symmetrical development. The healthy skin was not pruritic. In all, some 60 lesions were counted. The site of greatest aggregation was the hands and feet. In a few places could be seen cicatricial points. The question arose, were these lesions sequelæ of the conditions described as blisters? The patient stated positively that the original tumors had never disappeared. Even the ulcerated and cauterized patches would return. During 16 months of observation, there had been no new developments. One large, hickory-nut sized tumor, which had been removed, recurred in the original spot. The cicatrices were few and pea-sized, disproportionate to the existing lesions. The tumors were not painful, though the pruritus continued. The patient has taken mercury, arsenic and the iodide of potassium without affecting the lesions after 16 months.

Sections of the tumors had been examined by Dr. Heitzmann. There was found a hyperplasia of the epithelium and layers of the connective tissue. The rete was increased and the papillæ were enlarged and branching with narrowed capillaries. In the derma, were nests of visibly enlarged inflammatory elements in all stages of transition. The deeper layers of the derma were built up of very close bundles of connective tissue. The inflammatory process had chiefly affected the upper layers of the derma. The reader showed some micro-photographs of the sections made.

DR. HEITZMANN remarked that the upper layers exhibited the sole evidences of the process, the lower being unchanged. The disease was inflammatory, and not a new growth, one, however, with which he was not acquainted. The disease was certainly unique. The sections had been well made, Schultz's "thorns" showing very clearly. It was a scar-like disease of the thin and branching papillæ.

DR. L. A. DUHRING then read a paper entitled, SUPPLEMENT TO A CASE OF INFLAMMATORY FUNGOID NEOPLASM. The reader proceeded to give the full subsequent history of the patient presented by him before the Association at its last meeting, an account up to that date having been then read and subsequently published in the *Archives of Dermatology*, January, 1879. There had been a fatal termination, and the history of the entire symptoms during the last illness was graphically detailed with a full account of the post mortem section, and of the microscopical examination of several of the cutaneous tumors, as well as of several portions of the viscera and of a somewhat singular growth found in the bladder. The paper was illustrated by a new lithograph of the face of the patient, taken while she was living, and, when compared with that previously exhibited by the author, it furnished an exact picture of the changes effected by the malady since the last report. The sections subsequently made were also exhibited, and a portrait of the similar case described by Geber, was shown in colors.

In the interesting discussion which ensued, DR. HEITZMANN was strongly of the opinion, formerly expressed by him regarding this case, that the growth was a sarcoma, and he pointed to the fatal result which he had predicted from the microscopical appearances alone, as confirming this view. He praised the author for the exceedingly careful and accurate description given by him of the clinical and other features of the case.

On the other hand, a number of members pointed to the fact that the author had chosen a title for his two reports which was purely descriptive and not necessarily exclusive of sarcoma. Again, even on the hypothesis that the disease was really a sarcoma, it was claimed by several that the disease described by Dr. Duhring, was so different in its singular clinical history and microscopical features that it was certainly right and necessary to characterize and set these by themselves. As to the prediction made by Dr. Heitzmann, it was stated that an examination of the lady, without the aid of the microscope would have readily led, and in fact did lead, to the same conclusion.

DR. J. N. HYDE then read a second paper entitled, ON A VARIETY OF MOLLUSCUM VERRUCOSUM PRESENTING CERTAIN UNUSUAL FEATURES. The author described a case of molluscum observed by himself presenting certain rare peculiarities; notably, a tendency to recurrence of the lesions which were numerous; their bleeding when wounded; the absence of a central punctum and of sebaceous, milky, curdy or lobulated contents; the occurrence of pigmentary staining in the site of the lesions after disappearance; the absence of subjective sensation; and the vesicular aspect of the bodies. Opinions were read from Prof. Kaposi, of Vienna, to whom a photograph and minute description of the features of the case had been sent, and also from Dr. S. C. Busey, of Washington, who had been interested in the question of the telangiectasic or lymphangiomatous origin of the disease. The reader exhibited an oil painting of the patient, which represented the molluscous bodies at their full development, together with photographs of the case, and drawings of the appearances presented on microscopical examination of sections of the tumors. The conclusions of the paper were as follows:

"1. The case described is one exhibiting certain rare and peculiar features, and cannot, therefore, be made to serve as a basis for generalization. It is merely a contribution to the literature of the subject.

"2. Considering it, nevertheless, in connection with certain other cases, especially those described by Mr. Hutchinson, of London, it would seem to be reasonable to admit, that there is a variety of molluscum whose characteristics differ in a marked degree from those first observed by Bateman and since studied by later investigators. These characteristics are, briefly, an extraordinary multiplicity of small, pointed, white-capped, more or less solid lesions, resembling vesicles in their external appearance, generally destitute of milky or fluid contents, often without a central depressed orifice, and, at times, intermingled with other lesions, whose characteristics point unmistakably to the sebaceous origin of the disease.

"3. To the objection possibly brought against the conclusion last stated, that the lesions in question are merely incomplete and undeveloped forms of molluscous bodies, it may be responded that in the case here described, there were several recurrences of the disease, none influenced by either external or internal treatment, each accompanied by an abundant and copious development of the eruption, under circumstances particularly favorable to the growth of typical mollusca.

"4. The term molluscum verrucosum, proposed by Kaposi, properly designates the clinical features of the lesions to which the name has been limited in this sketch; and, in any view, is useful in distinguishing all the wart-like varieties of molluscum sebaceum, from those typical lesions to which the title of contagiosum was formerly applied.

"5. As between those authors who hold that even the atypical or wart-like forms of molluscum, originate in disorders of the sebaceous glands, and the authors who oppose this view, the case here described would point to an origin from protoplasmic bodies in the rete by proliferation, rather than to a disorder originating primarily in the sebaceous glands.

DR. ATKINSON remarked that he had observed a case of probably similar character, in the person of a young mulatto woman. Unfortunately the patient had soon after escaped from observation.

Adjourned at a late hour.

THURSDAY MORNING SESSION.—DR. J. C. WHITE read a paper entitled, ETIOLOGY. Referring to the views of different authors respecting the etiology of cutaneous disorders, the reader showed that, as a result of superstition or ignorance, the skin had been held guiltless of its own disorders, and had been made the battle-ground upon which were displayed the effects of disorders of all other parts of the body. Popular names of diseases of the skin embodied this belief, and "peccant humors," "efflorescences," "breaking out," etc., lay at the root of the danger supposed to arise from interference with skin disease, resulting in their "striking in," being "driven in," etc. But: 1. The skin enjoyed an inherent independence of other organs. 2. Its pathological processes were identical with those taking place elsewhere. 3. The same methods of observation and reasoning were applicable to it, as to other parts. Upon the first point, the reader stated that those who denied the autonomy of the skin, chiefly the French school, presented arguments in favor of their doctrines which were founded purely on theory, and in favor of which no positive fact was presented. The efforts to combat the so-called "dartrous diathesis" were suggestive of the attempt to exorcise spirits. In England, gout was of frequent occurrence, and the doctrine of a gouty diathesis was there held to account for the existence of skin diseases; but in this country, where gout was rarely encountered, the same skin diseases existed, and with nearly the same frequency. In malarial districts, malaria was esteemed to be the cause of skin troubles, which yet did not fail to manifest themselves in non-malarial districts. It was charged that the blood was disordered in many cases, but where was the satisfactory evidence that actual morphological changes had there taken place? Where, the proof that these changes, if established, had a direct and etiological connection with the skin disorder? Where, the great mass of collected facts necessary to place the proposition on the basis of clinical demonstration? The same charge had been brought against the urine, but in disorders of the skin, the changes in that fluid were trivial, and those which were undeniably to be found in the course of other disorders. He did not esteem lightly the evidence afforded by chemistry, nor did he fail to recognize the relation between the kidneys and the general conditions of the body; but with respect to the precise conditions which it was sought to establish, he thought applicable here the aphorism of Huxley, that there was "no approximation to a demonstration."

Upon the third point, the reader continued by briefly referring to the several viscera, which were esteemed to be efficacious in the production of skin diseases. Of the brain and nervous system, it was said that it had become fashionable of late to refer to them as the sources of all diseases. He admitted that delicate nerve fibres had been recently traced as far as the rete, and that it was well known that certain efflorescences were the result of what was called reflex action and nervous irritation. But what did the terms, "faulty innervation," "neurosis," etc., mean, beyond the mere fact that the blood vessels were under the control of the nerve fibres in the skin, as in other parts of the body. Nervous pathology lent but little support to dermatology. Did we look for a great development of skin diseases in insane asylums? [Laughter.] As for the lungs, consumptives were not known to suffer from disorders of the skin to a greater extent than other patients. The doctrine that parasites especially flourished upon such skins, was probably the result of the fact that persons with diseases of the chest exposed its surface to the examination of physicians more frequently than others. In 40 children affected with tinea in school, he had found as great exuberance of the vegetation upon the robust as upon the weak. So, too, respecting the stomach. While it was true that urticaria resulted from certain articles of diet, there was no basis for the general opinion that acne and eczema depended upon gastric disorder. Acne and dyspepsia were two common affections, and must often necessarily co-exist. For two years he had carefully examined every patient affected with acne as to the existence of dyspepsia, and had found but a small percentage of them suffering from any gastric disorder. In similar terms the reader discussed the agency of the heart, the liver, the kidneys and the reproductive organs, denying to each any special agency in the causation of cutaneous maladies, fully recognizing, however, the intimate relations between the genital organs and the skin, as evinced by study of comparative anatomy, in the pigment and tissue changes connected with the season of rut, etc.

DR. HEITZMANN followed with a high encomium of the paper which had been read. He fully endorsed the views of its author, and expressed his pleasure that they had been presented to the Association by its first president, the oldest of American dermatologists.

DR. FOX dissented from some of the opinions expressed, declaring that after his experiences in Vienna, where he had been taught the doctrines of the German school, and his studies in London and Paris, his views had undergone a change. He believed in the sympathy of all the organs of the body, and thought that even in functional disorders of the internal organs, there were skin changes.

DR. SHERWELL enquired whether the reader would not employ internal treatment in urticaria, and was answered that the treatment of skin diseases was not under discussion, but only the question of etiology.

DR. BULKLEY remarked that he would be willing to rest the argument upon the admissions which had been made by Dr. White in the paper read. Enough had been said to establish the fact that internal causes did operate to produce diseases of the skin. The reader had, however, ignored the exanthemata, syphilis and the influence of diet and hygiene in the production of effects.

DR. TAYLOR expressed his belief in the value of internal treatment of many cutaneous disorders.

DR. FOX recurred to the subject of acne in connection with gastric disorders. Being asked by DR. HYDE, whether he had not treated patients with acne who had never suffered from dyspepsia, etc. He admitted that he had.

DR. WIGGLESWORTH believed that there was some misconception of terms as employed. The treatment of the disease and the treatment of the patient were two very different things. In point of fact, the skin was either an external or an internal organ of the body, according to its temporary situation, and internal treatment was actually local treatment. All treatment is, to a certain extent, local. Take the case of a military garrison.

They succumb to the enemy only when sufficiently reduced in their defensive power to be unable to resist attack. A laundress when her health is good, does not suffer in her hands from the external irritation to which they are exposed at the tub. But once let there be a condition of unstable equilibrium, and the processes of waste and repair are disturbed, her epithelium, removed by friction, etc., is imperfectly restored. She becomes the victim of the " washerwoman's itch."

DR. R. W. TAYLOR then read a paper entitled, ON THE NATURE OF SYPHILIS. He discussed the various theories which have been put forward in connection with this subject, dissenting from the view of Hutchinson that syphilis ends with its secondary period, from the view of Després that syphilis is a purulent diathesis, there being no pus in the secretion of the initial sore, and from the views of Otis, expressed in 1871, as to the lymphatic origin of the disease.

" Syphilis is a disease of the connective tissue, and not primarily of the lymphatics or of the blood-vessels, although the blood may be temporarily modified and may be the vehicle of contagion. The secretions of syphilitic lesions are found to consist of a serous fluid containing numerous shining granules or molecules, which are masses of protoplasm or germinal matter, holding the contagious properties of syphilis. These microscopic bodies are probably taken into the circulation by the lymphatics, and conveyed over the body. The fact that serum alone does not convey the syphilitic poison goes to prove that the corpuscles hold the contagious material.

"In the secondary period of syphilis these cells are very numerous, and the body may be covered with papules and tubercles composed of them. As the disease wanes, these lesions become more localized and fewer in number, and the blood is less contagious. Finally, these cells may be limited to a few gummous tumors; the blood no longer carries the molecules, and it loses its contagious properties. The cells no longer have a tendency to reproduction, which characterizes them in the early stages, but rather degenerate. Hence we consider the blood and the secretions in tertiary syphilis innocuous. . . . . The periods of latency observed in the course of syphilis are of interest, and may perhaps be explained in the following way: Each outburst is attended by the development and multiplication of the peculiar cells, which run their course and are fully absorbed. Some remain, and after a time are excited by unknown causes to activity. Thus repeated exacerbations may occur, each one depending upon the multiplication of cells remaining from a previous outburst. But each relapse is less active and less prolonged than its predecessor, until perhaps only one nodule, and that composed of effete cells, may remain. The disease is thus cured."

DR. ATKINSON declared that he could not imagine cells which were too old to reproduce themselves! Did age depress the vitality of the cellular elements of the body to that extent? We have, furthermore, proof that all parts of the body are not infected with syphilis when the disease exists in an individual.

DR. HEITZMANN inquired whether the reader had intended to convey the idea that the carriers of contagion in syphilis, were granules.

DR. TAYLOR said that, in the unirritated lesion, there was no pus, but that shining molecules of protoplasm could be there detected which differed from the appearances presented by the chancroid ulcer.

DR. HEITZMANN:—"Molecules of protoplasm" is an unheard of term. Do these "molecules of protoplasm" carry the disease?

DR. TAYLOR:—They deposit and carry the disease.

DR. HEITZMANN:—What proof have you that the fluid part does not carry the disease?

DR. TAYLOR:—That is assumed since it can be demonstrated that the serum of the initial lesion is not inoculable. The albumen is coagulated.

DR. HEITZMANN:—"Assumed" is the word. All this is cheap theorizing! Who has heard of protoplasmic cells and molecules? Give us a demonstration of facts. Thus Lostorfer described his "corpuscular elements." What we need is fact and observation. The whole thing is at present a puzzle, except that we know mercury is good, and the best smearer of it, is the best man to treat the disease.

DR. TAYLOR responded that his opinions were not wild theories. That they were virtually the views of Cornil, Wegner, Wagner, Lancereaux and other eminent syphilographers, who had for many years studied the evidences of syphilis with the microscope and with as much care as any of those present.

The last paper read was a short communication by DR. W. A. HARDAWAY, entitled A SIMPLE METHOD OF OBLITERATING VARICOSE VESSELS IN ROSACEA. He employed a No. 13 cambric needle, attached to the negative pole of a galvanic battery, the patient holding the sponge electrode in the palm of the hand. Six to eight feebly-charged cells were required. Few vessels bled during the operation; indeed the column of blood could be seen to run up the lateral channels, probably in consequence of the evolution of gas during the process. Sometimes the vessel itself was punctured, but it was not necessary to hit this in every case. The operation was followed by no unpleasant results. It was not nearly so difficult a task as the obliteration of the hair papilla. The needle was inserted to the depth of from one and one-half to two millimetres, and kept in contact for a few seconds. In some cases, he had failed on the first attempt, and had succeeded in the second.

DR. WHITE stated that, in all the cutting and gashing operations practiced by him for the relief of these cases, he had been greatly disappointed, in consequence of his want of success.

DR. HYDE remarked that the question of the success of all such operations depended upon the extent of the telangiectasis. If there were superficial vessels only, enlarged and tortuous, then there might be a prospect of good results from either division or acupuncture with electrolysis. If, however, there were blood-vascular channels of any depth below the surface, the operation would almost surely prove to be a failure.

DR. HARDAWAY concluded by repeating that the use of a fine needle was essential in producing his results.

The Association, after transacting further business not of a scientific character, and listening to a brief concluding address by the President, adjourned.

The following specimens were then informally exhibited :—
Microscopical slides representing various forms of disease of the skin, by Drs. Duhring, Hardaway, and Van Harlingen; a drawing of a case of maculæ, symmetrically disposed on each side of the spinal column, and hairs from a case of trichorhexis nodosa, from a patient presented to the British Medical Association, by Dr. Duhring; photographs of a case of elephantiasis arabum from Indiana, and of severe lupus vulgaris in Iowa, by Dr. Hyde.

At an informal gathering of members in the amphitheatre of the New York Hospital, on Thursday afternoon, the following rare and interesting cases of skin disease were also exhibited:—Urticaria pigmentosa; lichen planus; "scleroderma, hemiatrophia facialis and alopecia areata;" tubercular leprosy with rare pigmentation; three cases of lupus erythematosus and lupus vulgaris; two syphilitic cases; carcinoma of the skin; lichen ruber; and scrofuloderma with failure of osseous development beneath the lesions.

